# Unlocking the Potential of Liquid Biopsy: A Paradigm Shift in Endometrial Cancer Care

**DOI:** 10.3390/diagnostics15151916

**Published:** 2025-07-30

**Authors:** Nannan Gui, Chalong Cheewakriangkrai, Parunya Chaiyawat, Sasimol Udomruk

**Affiliations:** 1 Department of Obstetrics and Gynecology, Faculty of Medicine, Chiang Mai University, Chiang Mai 50200, Thailand; nannan_gui@cmu.ac.th; 2 Department of Obstetrics and Gynecology, Faculty of Medicine, Affiliated Hospital of Youjiang Medical University for Nationalities, Baise 533000, China; 3 Center of Multidisciplinary Technology for Advanced Medicine (CMUTEAM), Faculty of Medicine, Chiang Mai University, Chiang Mai 50200, Thailand; parunya.chaiyawat@cmu.ac.th (P.C.); sasimol.ud@cmu.ac.th (S.U.)

**Keywords:** liquid biopsy, endometrial cancer, biomarkers, non-invasive, personalized medicine, early diagnosis

## Abstract

Endometrial cancer is one of the most prevalent gynecologic malignancies in developed countries, with its incidence steadily increasing each year. Early diagnosis is crucial for a favorable prognosis; however, certain patients experience recurrence and distant metastasis after surgery, similar to advanced cancer patients, with limited treatment options. Therefore, effective strategies for early screening, diagnosis, predicting local recurrence, and guiding rapid treatment interventions are essential for improving survival rates and prognosis. Liquid biopsy, a method known for being non-invasive, safe, and effective, has attracted widespread attention for cancer diagnosis and treatment. Although its clinical application in endometrial cancer is less established than in other cancers, research on biomarkers using liquid biopsy in endometrial cancer patients is currently in progress. This review examines the latest advancements in non-invasive biomarkers identified through liquid biopsy and provides a comprehensive overview of their clinical applications in endometrial cancer. Additionally, it discusses the challenges and future prospects of liquid biopsy, offering valuable insights into the diagnosis and personalized treatment of endometrial cancer.

## 1. Introduction

Endometrial cancer (EC), a malignant epithelial tumor of the uterus, ranks sixth among the most common cancers in women from developed countries [1]. Despite our expanding understanding of EC over time, both incidence and mortality rates continue to rise steadily [1,2]. Prognosis in EC largely depends on the tumor stage at diagnosis. The 5-year survival rate for Stage I patients is approximately 92% but declines significantly in more advanced stages, dropping to 74%, 48%, and 15% for Stages II, III, and IV, respectively [3]. Current EC guidelines recommend surgery for early-stage, low-risk cases, and a combination of surgery and postoperative adjuvant therapy for high-risk or advanced cases [4]. Notably, since the International Federation of Gynecology and Obstetrics (FIGO) incorporated molecular typing into the EC staging system in August 2023 [5], molecular profiling has become an increasingly valuable tool for guiding treatment decisions. However, despite these advances, the prognosis for patients with advanced or recurrent EC remains poor [6]. This may be attributed to the limited sensitivity of current imaging techniques in detecting early metastases, while molecular subtyping does not aid in early diagnosis. Within molecular subtype-guided therapy, patients with the copy-number low (NSMP) subtype, particularly those who are estrogen receptor-negative, tend to have a significantly worse prognosis [7,8]. Notably, patients with P53-abnormal EC have the poorest outcomes; even without chemotherapy, approximately 40% of those with TP53 mutations remain disease-free for five years [9]. These challenges highlight the ongoing need for improved risk stratification and guidance in adjuvant therapy. Therefore, finding more reliable tools and sensitive biomarkers is crucial for early diagnosis and personalized treatment. Recently, liquid biopsy has gained significant attention as a promising tool for precision medicine, cancer diagnosis, and therapy [10]. This technique involves analyzing non-solid biological materials such as blood, urine, cervical fluid, uterine aspirate, and peritoneal lavage fluid. Compared to conventional tissue biopsies, liquid biopsies are non-invasive, repeatable, and allow for real-time monitoring of treatment response and disease progression. They also overcome challenges related to anatomical sampling, patient age, cost, reproducibility, and clinical complications. Furthermore, they offer a more comprehensive view of heterogeneous and multifocal metastatic tumors [11]. Although the use of liquid biopsy in EC is still developing compared to other malignancies, research in this area is expanding. It is expected to play an important role in early detection, risk assessment, treatment selection, and real-time disease monitoring in EC. While recent reviews have primarily focused on blood-based biomarkers in liquid biopsies for EC [12,13,14], this review takes a broader perspective. It examines studies that utilize a variety of biosources, including both blood-based and non-blood-based specimens from EC patients. Additionally, we explore the advantages, limitations, and future developments of liquid biopsy technologies, offering new insights and directions for the personalized diagnosis and management of EC.

## 2. Methods

For this review, we conducted a PubMed (MEDLINE) search in February 2025 for full-text, English-language articles on liquid biopsy in endometrial cancer published between January 2019 and January 2025. The keywords used were “liquid biopsy,” “endometrial cancer,” and “clinical relevance.” The initial search identified 720 titles, which were screened for relevance to the topic. Two reviewers independently assessed the titles and abstracts (360 each), and any discrepancies were resolved through discussion to reach consensus. After the initial screening, 120 articles underwent full abstract review. Articles were included if they met the following criteria: focused on the application of liquid biopsy in endometrial cancer, were published in a peer-reviewed journal, and provided clinical or experimental data on liquid biopsy for diagnosis, prognosis, or treatment monitoring in endometrial cancer. A total of 82 articles were selected for detailed analysis based on these criteria. Additionally, foundational studies published before 2019 were included if they were frequently cited in recent research or were essential for understanding key concepts, methods, or biomarkers relevant to liquid biopsy in endometrial cancer. EndNote 21.4 for Windows was utilized to manage and cite references throughout the manuscript.

## 3. Biological Components of Liquid Biopsy

Liquid biopsy targets can be broadly categorized into two groups based on their biological nature. The first includes cell-free molecules such as proteins, lipids, carbohydrates, metal ions, nucleic acids, and small metabolites. The second consists of cellular or subcellular components, including extracellular vesicles, circulating mitochondria [12], circulating tumor cells, peripheral blood mononuclear cells (PBMCs), circulating cancer-associated fibroblasts (CAFs) [13], and tumor-educated platelets (TEPs) [14]. Detection methods vary depending on the specific target type in the sample.

### 3.1. Circulating Tumor Cells

Circulating tumor cells (CTCs), originating from primary solid tumors or metastatic sites, enter the peripheral circulation through processes such as cellular invasion, matrix degradation, and angiogenesis. Only a small subset of CTCs—those with stem cell-like properties or epithelial–mesenchymal transition (EMT) features—can survive and migrate. Most CTCs are quickly eliminated by the immune system or destroyed by shear forces [15]. CTCs have a very short half-life, ranging from 1 to 2.4 h [16], and are extremely rare, with only 0 to 28 cells typically detected in 7.5 mL of blood [17]. Their high heterogeneity leads to variable surface biomarker expression [18], making detection and the development of standardized treatment guidelines challenging. Although several clinical trials in breast cancer and colorectal cancer have validated CTC enumeration as both a prognostic biomarker and a potential tool for guiding therapy, its predictive value for treatment selection still requires further validation to establish definitive clinical utility beyond prognosis [19,20,21,22].

CTC detection typically involves three main stages: enrichment, detection, and analysis. Enrichment methods use physical properties—such as density, size, and electrical charge—biological approaches based on specific binding to cell surface antigens, or a combination of both. CTCs are most commonly captured using immunomagnetic enrichment or size-based microfluidics. The clinical gold standard is the CellSearch^®^ System (Menarini-Silicon Biosystems, Huntingdon Valley, PA, USA), which immunomagnetically enriches EpCAM-positive CTCs using anti-EpCAM ferrofluid beads, followed by cytokeratin staining and CD45 exclusion; it received FDA approval for metastatic breast cancer [23], colorectal cancer [24], and prostate cancer [25]. Label-free microfluidic platforms, such as the Parsortix^®^ PC1 System (ANGLE North America, Inc., King of Prussia, PA, USA), trap CTCs by size (≥8 µm) and low deformability, harvesting intact cells for downstream assays; it obtained FDA approval for metastatic breast cancer patients [26,27]. Emerging platforms (negative-depletion immunomagnetic, inertial microfluidics, vortex trapping, ligand- and aptamer-based, label–capture–release) aim to capture diverse CTC subtypes [28,29,30,31,32,33].

Molecular detection methods for CTCs include (i) nucleic acid analysis: Techniques such as fluorescence in situ hybridization (FISH) [34], microarrays [35], and Polymerase Chain Reaction (PCR)-based techniques [36], as well as sequencing-based techniques [37], are employed to detect genomic DNA or RNA signatures from CTCs in various body fluids. While these methods offer high sensitivity, their accuracy can be affected by background substances, including non-specific DNA/RNA, PCR inhibitors, and cross-hybridization, potentially leading to false positives or reduced accuracy. (ii) Protein analysis: This approach focuses on identifying and characterizing surface or intracellular proteins of CTCs using techniques such as microfluidic technology [38] and enzyme-linked immunospot (ELISPOT) [39,40]. While this approach reduces extensive or invasive manipulation of the target cells, thereby minimizing potential cellular interference, it can be time-consuming. (iii) Cellular function analysis: Culturing CTCs in vitro allows for the study of their proliferation, transformation, and invasion capabilities. Although this method offers high specificity, it is prone to cultivation failure due to the low viability and heterogeneity of CTCs, as well as factors such as initial cell count, cancer type, and culture conditions [41].

### 3.2. Circulating Tumor DNA and Cell-Free DNA

Extracellular DNA fragments known as cell-free DNA (cfDNA) are discharged into the bloodstream by a number of cellular processes, including necrosis, apoptosis, and secretion [42]. These fragments, which may be single- or double-stranded, gain stability in circulation by binding to cell membranes and extracellular proteins, protecting them from nuclease-mediated degradation and rapid clearance. Circulating tumor DNA (ctDNA), released by cancer cells, reflects the tumor genomes from various sites, including primary tumors, CTCs, and metastases. Unlike a single tissue biopsy, ctDNA captures the molecular heterogeneity of cancer. It contains key genetic alterations found in tumor tissues, such as chromosomal rearrangements, point mutations, copy number variations, epigenetic modifications, insertions, and deletions [43]. In cancer patients, ctDNA levels range from 0.01% to 10% [44]. It is typically shorter than cfDNA—about 134 to 144 base pairs—with a half-life of approximately 114 min, making it a valuable tool for real-time tumor monitoring and assessing treatment response [44,45].

The concentration of ctDNA in plasma is relatively low, but due to less contamination from white blood cell DNA, it is typically the preferred choice in clinical tests [46]. PCR-based methods, including quantitative PCR (qPCR) and droplet digital PCR (ddPCR), are commonly used for detecting cfDNA and ctDNA due to their cost-effectiveness and high sensitivity. However, there are notable differences between these two techniques. qPCR is a traditional method that quantifies DNA through amplification cycles, with results represented as a relative quantity of the target. In contrast, ddPCR is a more advanced technique that partitions the sample into thousands of droplets, performing PCR in each droplet, and provides an absolute quantification of the target DNA without relying on standard curves, making it more precise and less susceptible to PCR efficiency variations [47]. The limited ability of both techniques to detect multiple mutations has led to the growing use of Next-Generation Sequencing (NGS) techniques for more comprehensive analysis, such as CAncer Personalized Profiling by deep Sequencing (CAPP-Seq) [48].

Epigenetic modifications, particularly ctDNA methylation patterns, have emerged as important biomarkers for cancer diagnosis and prognosis. Detection methods for ctDNA genomic regions and genome-wide methods enable comprehensive analysis [49]. PCR-based assays such as methylation-specific PCR (MSPCR) [50] and droplet digital MSPCR (ddMSPCR) [51], as well as target bisulfite sequencing, are examples of targeted approaches. Whole-genome bisulfite sequencing (WGBS) [52], TET-assisted pyridine borane sequencing (TAPS) [53], reduced representation bisulfite sequencing (RRBS) [54], and Infinium methylation arrays (HM450, HM850) [55] are examples of technologies used in genome-wide approaches, which include both site-specific and region-wide analysis. Advanced techniques, such as cfMeDIP-seq and nanopore sequencing, further enhance methylation profiling and are especially well-suited for analyzing the low-abundance, fragmented ctDNA found in liquid biopsies [56].

### 3.3. Circulating Tumor RNA and Cell-Free RNA

Cell-free RNA (cfRNA), including circulating tumor RNA (ctRNA) derived from cancer cells, consists of various types, such as circular RNA (circRNA), microRNA (miRNA), and long non-coding RNA (lncRNA). Unlike cfDNA, which is primarily released through passive mechanisms, cfRNA can enter the bloodstream through both passive processes and active secretion by cells [57]. Although cfRNA has a plasma half-life of just 15 s, its stability is enhanced through interactions with proteins [58], proteolipid complexes, and extracellular vesicles [59]. Detection methods for cfRNAs and ctRNAs include PCR-based techniques such as quantitative reverse transcription PCR (qRT-PCR) and reverse transcription PCR (RT-PCR), as well as sequencing methods. Among these, qRT-PCR stands out for its ability to quantify cfRNA targets with high sensitivity and reproducibility [60], similar to qPCR for cfDNA, but it specifically amplifies complementary DNA synthesized from RNA templates. qRT-PCR is commonly used for analyzing specific cfRNA biomarkers, whereas NGS allows for a more comprehensive analysis of cfRNA profiles.

Individual cfRNA profiles vary significantly [61], and the lack of standardized clinical protocols may contribute to false positive and false negative results in clinical practice [62]. Consequently, researchers have tried to focus on RNA methylation. Common techniques for detecting RNA methylation include antibody-based immunoprecipitation combined with deep sequencing, mass spectrometry (MS), thin-layer chromatography, radioactive isotope incorporation, and bisulfite modification followed by sequencing [63]. Among these, methylated RNA immunoprecipitation sequencing (MeRIP-seq) remains a key method for identifying RNA methylation modifications [64]. While the potential of cfRNA in liquid biopsy is promising, it is important to note that its clinical implementation is still in its early stages, and many challenges, such as the standardization of detection methods and the interpretation of cfRNA profiles, remain to be addressed.

### 3.4. Extracellular Vesicles

Extracellular vesicles (EVs), including exosomes, microvesicles, and apoptotic bodies, are distinguished by their size, surface properties, biogenesis pathways, and molecular content. Exosomes, first discovered in the late 1960s [65], are the tiniest nanoscale EVs, typically ranging from 40 to 200 nm in diameter and having a density of 1.13 to 1.18 g/mL [66]. Produced by nearly all cell types under both healthy and diseased conditions, these vesicles facilitate the transfer of proteins, lipids, and nucleic acids, playing a key role in intercellular communication [66]. In cancer, EVs are involved in nearly every stage of disease progression, including the transformation of normal cells [67], tumor growth [68], angiogenesis [69], modulation of tumor microenvironment [70], invasion and metastasis [71], drug resistance [72], and EMT [73]. As such, EVs are considered promising candidates for cancer diagnosis, prognosis, and the development of therapeutic biomarkers.

Methods for EV enrichment and detection leverage their inherent properties, such as size, density, surface composition, and precipitation behavior. Currently, commonly used techniques include ultracentrifugation, ultrafiltration, precipitation, immunoaffinity capture, and lipid-based isolation [74]. Advanced approaches—such as microbeads, microfluidic chips, and thermal methods—are also being explored to enhance enrichment efficiency. Traditional detection methods like enzyme-linked immunosorbent assay (ELISA) [75] and Western blot analysis [76] remain reliable. However, emerging techniques, including colorimetry [77], fluorescence [78], flow cytometry [79] electrochemical analysis [80], electron microscopy [81], nanoparticle tracking analysis (NTA) [82], and CRISPR/Cas-assisted methods [83], as well as single exosome detection [84], are enhancing the sensitivity and specificity of exosome research, particularly in the context of liquid biopsies.

### 3.5. Proteomics

Proteomics complements genomics, transcriptomics, and metabolomics by analyzing protein distribution, structure, interactions, and alterations within biological systems to offer a thorough understanding of biological processes [85]. Unlike the static nature of genomes, proteomes dynamically vary across different life stages and functional states. Early liquid biopsy efforts in cancer focused on identifying protein biomarkers in blood [49]. Although over a hundred biomarkers, such as HE4 for ovarian cancer and SCC for cervical cancer, are used for treatment monitoring and recurrence assessment, their effectiveness in early detection is limited by insufficient specificity and sensitivity [49], underscoring the need for more advanced diagnostic approaches.

Technological advancements have revolutionized proteomics, shifting from traditional moderate-throughput methods such as ELISA and CLIA to high-throughput techniques like antibody/antigen arrays, proximity extension assays (PEAs), reverse-phase protein arrays (RPPAs), and aptamer-based platforms [86]. MS now plays a central role, offering rapid protein sequencing, precise molecular weight determination, and quantitative detection of post-translational modifications [87]. In liquid biopsy analysis, MS is often integrated with liquid chromatography (LC), enzymatic digestion, and desalting, followed by electrospray ionization (ESI) and tandem MS scanning to enhance detection accuracy and sensitivity [88]. Recent advancements in MS instrumentation, including improved ion transmission efficiency and advanced noise-reduction algorithms, have significantly boosted single-cell and targeted proteomics.

While high-throughput methods support large-scale profiling, single-cell proteomics technologies address cellular heterogeneity. Techniques like mass cytometry for CTC immunophenotyping [89], microfluidics-based CTC isolation [90], and single-cell Western blotting [91] enable detailed protein expression analysis at the single-cell level, providing critical insights into cancer cell heterogeneity. However, antibody-based detection still faces limitations in specificity and throughput, necessitating further optimization to reduce cross-reactivity and enhance multiplexing capabilities.

### 3.6. Metabolomics

Metabolomics, systematically defined by Nicholson et al. in 1999 [92], focuses on the comprehensive analysis of low-molecular-weight metabolites (<1500 Da) using advanced spectroscopic, electrochemical, and computational techniques. Because metabolites rapidly respond to microenvironmental changes, they offer dynamic insights into physiological and pathological states, making metabolomics a highly sensitive approach for biomarker discovery [93]. As a non-invasive tool in liquid biopsy, metabolomics enables the identification of disease-associated biomarkers in biofluids; however, its clinical specificity still requires further validation to account for potential confounding factors.

Metabolomics analyses typically follow two complementary strategies: non-targeted approaches, which explore global metabolite profiles for hypothesis generation, and targeted approaches, which quantitatively assess predefined metabolites in a hypothesis-driven manner [94]. Key analytical platforms for metabolomics include nuclear magnetic resonance (NMR) [95] and MS. NMR offers rapid, non-destructive analysis with high reproducibility; however, conventional ^1^H NMR is limited in sensitivity and spectral resolution, especially for low-abundance metabolites or complex mixtures [95]. Emerging NMR technologies—such as two-dimensional spectroscopy [95] and cryogenic probe-assisted ^13^C detection [96]—show promise in addressing these limitations.

MS-based technologies, including gas chromatography–MS (GC-MS) and liquid chromatography–MS (LC-MS), offer broader metabolite coverage and superior sensitivity [97]. LC-MS is ideal for non-volatile compounds, while GC-MS is suited for volatile metabolites. Recent advancements, such as ultra-performance LC-MS/MS (UPLC-MS/MS) [98] and nanoparticle-enhanced laser desorption/ionization MS (NPELDI-MS) [99], have further improved detection limits and ionization efficiency, enabling high-throughput metabolomics.

## 4. Application of Liquid Biopsy in EC

Liquid biopsies are increasingly recognized as valuable tools in EC management, with applications spanning early detection, prognosis, recurrence monitoring, and therapy guidance. In the following discussion, original research articles published on PubMed between January 2019 and January 2025, focusing on the use of liquid biopsy in EC, will be compiled and presented in a summary table along with relevant commentary. This analysis will be organized from two perspectives: studies based on blood-derived samples and those utilizing non-blood-derived specimens.

### 4.1. Blood-Based Liquid Biopsy in EC

Blood is the primary and most significant source for liquid biopsy. While tumor heterogeneity poses a major challenge in tissue-based sampling, liquid biopsy using blood allows for a more comprehensive and dynamic assessment of EC patients (Table 1).

#### 4.1.1. Early Diagnosis

The utility of cfDNA and cfRNA in the diagnosis of EC has been demonstrated [104]. Compared to cfRNA, cfDNA is generally preferred by researchers due to its greater stability and the more advanced state of detection technologies. Researchers have investigated cfDNA from various perspectives. For instance, in terms of concentration, Gressel et al. [106] found that the median concentration of low-molecular-weight (LMW) cfDNA was significantly higher in EC patients compared to healthy controls. In contrast, Benati et al. [105] examined relative telomere length (RTL) in cfDNA and found EC patients had markedly shorter RTL than healthy individuals, with promising early diagnostic accuracy (AUC = 0.87). More commonly, studies focus on gene mutations [14,111] or DNA methylation [110], utilizing either PCR- based or sequencing technologies. For example, Beinse et al. [110] identified hypermethylation of the ZSCAN12 and OXT genes in the ctDNA of EC patients. Using ddPCR, they achieved high diagnostic sensitivity and specificity (both 97%), successfully detecting ctDNA in 14 of 31 plasma samples collected before surgery or chemotherapy, including cases from both early and advanced stages. These findings highlight the potential of ctDNA methylation analysis as a non-invasive and personalized tool for monitoring and managing EC.

Similarly, cfRNA is being explored as a potential diagnostic biomarker. Fan et al. [119] identified six miRNAs that were overexpressed in the serum of EC patients. The diagnostic performance of this six-miRNA signature yielded AUCs of 0.748, 0.833, and 0.967 in training, testing, and external validation cohorts, respectively. Moreover, the expression levels of miR-143-3p and miR-195-5p in tissues, as well as miR-20b-5p in serum exosomes, were consistent with their serum levels, further supporting their diagnostic relevance. In addition to verifying the potential of miRNAs as early biomarkers, Rostami et al. found that the association between EC and miRNA expression is modulated by factors such as body mass index, physical activity, and adherence to a Western diet [123].

An increasing number of studies have demonstrated that the plasma protein profiles or metabolomic features can aid in the early diagnosis of EC [127,128,129,135,136,139,140,142,145]. However, the specificity of these diagnostic approaches remains limited, underscoring the need to combine multiple biomarkers such as cfDNA, cfRNA, proteins, and metabolites to enhance accuracy [145,148]. With the advancement of artificial intelligence (AI), machine learning (ML) has been increasingly applied for biomarker screening and model development to enhance diagnostic performance [130,133,149]. For instance, Troisi et al. applied an ensemble machine learning (EML) algorithm to screen and detect EC in postmenopausal women, achieving an accuracy rate of 99% [133]. Despite these advances, interpreting the results from such “black box” models remains a key challenge for researchers moving forward. In terms of sample collection site, Francini et al. [102] offered a novel perspective. In their preliminary study on CTC detection during early-stage EC surgery, 80% of patients had detectable CTCs in the ovarian vein, whereas none were found in peripheral blood samples. This suggests that ovarian vein sampling may offer greater sensitivity for CTC detection. In contrast, Kodada et al. [111] identified DNMT3A and TET2 mutations in ctDNA from peripheral plasma that were absent in tumor tissue, indicating challenges in distinguishing tumor-specific mutations from age-related clonal hematopoiesis (ARCH). Their findings suggest that background noise in EC diagnostics might be reduced by analyzing ctDNA from non-blood specimens such as uterine lavage fluid.

#### 4.1.2. Recurrence Monitoring

Surgical removal of the tumor remains the primary approach in EC treatment, often followed by personalized adjuvant therapies based on postoperative assessment. Recurrence monitoring typically relies on radiographic imaging and serum tumor markers. However, these conventional methods often lack the sensitivity to detect minimal residual disease (MRD) or micrometastases after surgery. As a result, there is a critical need for more sensitive and specific biomarkers to enable early detection of recurrence and metastasis, which could significantly improve patient outcomes.

Emerging evidence suggests that ctDNA is a more accurate biomarker for monitoring EC recurrence. Feng et al. [108] used ddPCR to track common tumor-specific mutations, including PTEN, FAT4, ARID1A, and TP53, in the plasma of EC patients, achieving 100% sensitivity and 83.3% specificity. Their findings highlight ctDNA’s superior predictive value over traditional markers like CA125 and HE4. Recio et al. [113] further confirmed this through longitudinal ctDNA monitoring post-surgery. They demonstrated that patients with positive ctDNA at both the initial time point and longitudinally had significantly worse recurrence-free survival (RFS) (HR = 6.2; *p* = 0.0006 and HR = 15.5; *p* < 0.0001, respectively), with recurrence rates of 58% and 52%, compared to 6% and 0% in ctDNA-negative individuals. This suggests that postoperative ctDNA detection is a strong predictor of outcomes and a key risk factor for recurrence. Similar conclusions were drawn by Grassi et al. [109]. Likewise, Law et al. [103] used microfluidic technology to investigate CTC-related markers in gynecologic malignancies. Although the study encompassed various cancer types, the findings in EC were particularly notable. Markers such as PanCK, GATA3, HER2, and HE4 were consistently detected in preoperative samples. During follow-up, the reappearance of these markers was strongly associated with disease recurrence in EC patients, often preceding clinical symptoms. This suggests that these molecular markers could serve as early indicators of relapse, offering a critical window for timely intervention.

#### 4.1.3. Prognostic Prediction

Prognostic biomarkers help identify patients with aggressive tumors and offer valuable insights into long-term outcomes, independent of treatment strategies. Their main purpose is to predict prognosis and guide treatment intensity to improve survival in EC patients. With advancements in MS technology, many researchers have applied non-targeted metabolomics to identify prognostic metabolites in EC [132,134,143,144]. However, the predictive value of these metabolites is evident only when assessed in combination, as no highly specific individual markers have been identified. With growing insights into genomics, attention has increasingly shifted toward cfDNA and cfRNA. Studies show that cfDNA is associated with tumor size, disease stage and classification, invasive characteristics, cancer progression, lymphovascular invasion [104,107,109,111,112,115,116,117], and overall survival (OS), supporting its potential as a prognostic marker. Similarly, cfRNA holds promise [118,120,121]. For example, Wu et al. [120] found that reduced serum miR-204-5p levels correlate with lymph node metastases, while Shan et al. [118] proposed serum lncRNA DLEU1 as a prognostic biomarker linked to adverse clinical features and poor survival outcomes in EC.

In addition to free protein biomarkers in the blood [130,131], exosomal proteins are also being investigated. Song et al. [124] examined exosomal LGALS3BP as a potential biomarker for EC and found it significantly elevated in plasma exosomes from EC patients. Higher LGALS3BP levels were associated with increased cell proliferation, migration, angiogenesis, and poor prognosis. These findings highlight the potential of non-invasive markers from various sources, but further validation is needed to confirm their prognostic value and clinical utility in guiding treatment for EC.

#### 4.1.4. Treatment Guidance

Modern treatment options such as molecular targeted therapy and immunotherapy have improved survival in patients with advanced or metastatic EC. However, systemic anti-cancer treatments face challenges like primary resistance, lack of initial response, and acquired resistance. Additionally, tumor molecular profiles often change during therapy, necessitating continuous monitoring to evaluate treatment response and predict resistance. Blanc-Durand et al. [114] demonstrated that cfDNA profiling in advanced EC provided 89% molecular information and 87.5% concordance with tissue biopsies. This method guided targeted therapy in 16% of patients, yielding a median PFS of 7.7 months and a 56% response rate. These findings highlight the potential of cfDNA analysis to enhance personalized treatment strategies for advanced EC.

In CTCs from high-risk EC patients, Herrero et al. [101] identified overexpression of annexin A2 (ANXA2), which was associated with reduced OS and PFS. High-throughput screening identified daunorubicin as a potential therapeutic agent that inhibits ANXA2-driven metastasis by reducing the invasiveness of ANXA2-overexpressing cells. For non-endometrioid EC subtypes, Shen et al. used multi-omics analysis to identify proteins such as IL32 and GRB7, which are involved in key oncogenic pathways like MAPK signaling and cytokine–cytokine receptor interactions. These findings not only deepen our understanding of EC pathogenesis but also provide potential targets for molecularly tailored therapies.

### 4.2. Non-Blood-Based Liquid Biopsy in EC

Non-blood-based liquid biopsies offer a promising alternative to traditional blood sampling in EC. Blood-based biomarker detection can be challenging, particularly in early-stage tumors, due to the low abundance of circulating signals [150]. Alternatively, the close anatomical connection between the uterine cavity, lower reproductive tract, and urinary system presents new opportunities for biomarker discovery in EC [151]. Examples of these findings are detailed in Table 2 and Table 3.

#### 4.2.1. Urine Samples

Urine contains diverse components, including malignant cells, tumor-derived nucleic acids, peptides/proteins, endogenous metabolites, and secretory organelles [183]. Costas et al. [154] evaluated the utility of somatic mutation analysis in urine for non-invasive EC detection and molecular classification. Using NGS, they achieved a 100% mutation detection rate in EC cases, showing high concordance between urine and tumor samples, particularly when applying the Proactive Molecular Risk Classifier for EC (ProMisE) algorithm. These results suggest that urine-derived cfDNA, such as transrenal ctDNA (TR-ctDNA), may serve as a reliable biomarker for early EC diagnosis and prognosis. Similarly, Ritter et al. [153] identified miRNAs, such as miR-10b-5p and miR-205-5p, in urine, with miR-10b-5p demonstrating diagnostic potential in EC patients. While additional validation is required, these studies highlight the promise of urine-based miRNA profiling for non-invasive screening.

Beyond nucleic acid biomarkers, urine proteins and metabolites have also been investigated. Unlike Ritter et al. [153], who relied on case–control studies to detect protein concentration differences, Njoku et al. [155] applied machine learning to develop a diagnostic model using 10 urinary markers, achieving an accuracy of 0.92. Similarly, instead of analyzing urine metabolites alone, Chen et al. [156,157] combined serum and urine data, yielding an AUC of 0.922, demonstrating a valuable model-building approach for EC. Furthermore, Fu et al. [158] integrated metabolomics with transcriptomics, identifying differential metabolites and hub genes in urine associated with EC. This multi-omics strategy suggests that combining urine-based biomarkers with transcriptomic profiles could improve early EC detection.

As a distinct and promising sample source for liquid biopsy, urine offers a non-invasive, easily accessible, and disease-specific tool for EC diagnosis and management—potentially overcoming several limitations of blood-based sampling.

#### 4.2.2. Uterine Lavage Fluid and Uterine Aspirates

Uterine lavage fluid or uterine aspirates (UAs) represent a promising source for liquid biopsy due to their direct contact with tumors. Since Maritschnegg et al. [184] first detected shed EC cells in uterine lavage fluid, subsequent studies have further explored the diagnostic potential of these samples [185]. Casas-Arozamena et al. [159] provided the first comprehensive characterization of UAs, ctDNA, and CTCs. Their NGS analysis revealed genetic mutations in 93% of tumor samples, predominantly in genes such as PTEN, PIK3CA, and TP53. Notably, CTCs and ctDNA were found in 38.9% and 41.2% of cases, respectively, particularly among patients with high-risk tumors, suggesting their value as biomarkers for aggressive disease. Furthermore, they also demonstrated strong concordance between MSI results from UAs and cfDNA samples and those from traditional tissue, highlighting UAs as a viable tool for personalized monitoring and management [160].

Further supporting the utility of endometrial fluid analysis, Yang et al. [161] used real-time PCR to analyze specific miRNAs—miR-429, miR-146a-5p, and miR-183-5p—in endometrial fluid, underscoring their diagnostic potential for EC. This miRNA profiling offers a less invasive alternative to traditional diagnostic procedures, potentially improving early detection and intervention. However, uterine lavage collection can cause notable patient discomfort and requires specialized equipment and trained personnel, limiting its routine clinical use [186].

#### 4.2.3. Cervicovaginal Fluid and Cervicovaginal Lavage Fluid

Cervicovaginal fluid, which contains shed tumor cells originating from the lower reproductive tract, serves as an additional effective screening tool for minimally invasive sample collection compared to uterine lavage fluid, which has more limitations in its application. In recent years, researchers have carried out extensive studies on cervicovaginal fluid or cervicovaginal lavage fluid based on cytological analysis [163], somatic mutations [166], DNA methylation [167,169,170,171], proteomics [164,168,172,173], metabolomics [162,174], and multi-omics [165] approaches.

In contrast to the limited specificity of traditional cytology tests [163], growing attention has turned to DNA methylation as a more accurate diagnostic approach. Evans et al. [167] assessed the methylation status of ZSCAN12 and GYPC in cervicovaginal samples using the WID-qEC test. Compared to conventional ultrasound, WID-qEC demonstrated superior performance, achieving 92.1% specificity, 90.9% sensitivity, and an AUC of 94.3%. These results were further validated by Illah et al. [169], confirming WID-qEC as a highly sensitive and specific diagnostic method. Collectively, these studies suggest that cervicovaginal lavage offers a practical, minimally invasive alternative to traditional diagnostic procedures.

Beyond non-targeted approaches that screen proteins or metabolites for diagnostic [162,164,165,168,172] or stratification models [173,174], Pelegrina et al. [166] made a significant advancement by applying NGS to assess somatic mutations in cervicovaginal samples for non-invasive EC detection and molecular classification. The ClassEC test identified mutations in 73% of EC cases, with 80% specificity in clinician-collected samples and 90% in self-collected ones. Importantly, the test stratified EC into four molecular subtypes with distinct prognoses: POLE mutations were linked to favorable outcomes, while TP53 mutations predicted poor prognosis. This integration of molecular profiling with non-invasive sampling offers a promising alternative to traditional invasive diagnostics and represents a major step forward in personalized treatment for EC.

#### 4.2.4. Tampons

Tampons, as widely accepted and non-invasive intravaginal hygiene products, present a promising method for EC detection. Fiegl et al. [187] demonstrated that DNA methylation analysis of tampon-collected samples could distinguish EC from benign conditions with 100% sensitivity and 97.2% specificity in women aged 50–75, excluding CIN III and cervical cancer. Similarly, Bakkum-Gamez et al. [188] used tampons to collect vaginal pool samples and identified hypermethylation in nine genes in EC patients, achieving an AUC of 0.88, 76% sensitivity, and 96% specificity. This approach not only allows for convenient self-collection, improving patient compliance, but also enables repeated sampling for long-term monitoring in high-risk populations.

#### 4.2.5. Cervical Scrapings and Vaginal Swabs

In addition to tampons, vaginal swabs and cervical scrapings are valuable sources for molecular DNA testing in EC. These low-cost, minimally invasive methods can be easily incorporated into routine outpatient visits. Multiple studies have demonstrated high diagnostic sensitivity and specificity in detecting tumor-driver gene methylation through vaginal swabs [177,178,179,180]. Notably, Herzog et al. [178] evaluated methylation of the GYPC and ZSCAN12 gene regions in cervical, vaginal, and self-collected swab samples from patients with EC symptoms, reporting EC detection sensitivities of 100%, 90.1%, and 97.2%, respectively. This highlights the potential of self-sampling to support early detection while reducing the need for in-person visits. Interestingly, cervical lavage fluid also revealed abnormal methylation in these genes, validating the reliability of vaginal swabs and cervical smears. Furthermore, Kim et al. [176] successfully detected key gene mutations—such as PTEN, PIK3CA, TP53, and ARID1A—from genomic DNA in cervical smear samples with 100% specificity, aiding the optimization of ProMisE-based molecular classification for EC.

However, future research should prioritize pre-diagnostic sampling. Since most existing studies have focused on already-diagnosed individuals, earlier sampling could better reflect real-world diagnostic scenarios and reduce bias from tumor cell shedding during clinical procedures.

#### 4.2.6. Peritoneal Surgical Lavage Fluid and Peritoneal Fluid

Peritoneal surgical lavage fluid and peritoneal fluid have emerged as promising sources for detecting mutations and other genetic alterations associated with EC, offering diagnostic and prognostic value among various biopsy fluids. To validate the utility of peritoneal lavage fluid, Mayo-de-las-Casas et al. [181] used a highly sensitive qPCR method and found that, in EC cases with known hotspot mutations, cfDNA from peritoneal lavage had a significantly higher detection rate (47%) compared to plasma (10.5%). This indicates that peritoneal lavage may better reflect the tumor mutational landscape, particularly in early-stage disease. Similarly, Ayyagari et al. [182] evaluated sterol-O-acyl transferase 1 (SOAT1) and cholesterol ester (CE) levels in plasma, peritoneal fluid, and endometrial tissue from EC patients and controls. Elevated levels were observed in tumor tissues and peritoneal fluid from EC patients, while plasma levels were comparable between groups. The strong correlation between SOAT1, CE, and poor overall survival suggests these markers are linked to tumor aggressiveness and unfavorable prognosis. Thus, SOAT1 and CE may serve as prognostic biomarkers and potential therapeutic targets, with peritoneal fluid offering a more informative medium than blood for detection.

## 5. Future Directions and Prospects

Liquid biopsy is poised to become an essential component of EC management in the near future. Techniques involving cervicovaginal fluids, uterine aspirates, and circulating biomarkers—combined with genomic, proteomic, metabolomic, and multi-omics analyses—offer transformative potential for early detection and personalized treatment. These technologies promise to improve diagnostic accuracy, reduce reliance on invasive procedures, and enable more targeted therapeutic strategies. Early detection through such methods could significantly enhance patient outcomes by allowing timely, individualized interventions.

Integrating multi-omics approaches offers a comprehensive view of EC, uncovering potential therapeutic targets and providing deeper insights into tumor behavior, treatment response, and resistance mechanisms. Successfully translating these innovations into clinical practice will require close interdisciplinary collaboration among gynecologists, oncologists, geneticists, data scientists, and bioinformaticians. Such collaboration is key to developing integrated diagnostic platforms that improve diagnostic precision and enable personalized treatment strategies tailored to each patient’s molecular and clinical profile.

Recent advancements in imaging, histopathology, and molecular diagnostics emphasize the importance of an integrated approach that combines various testing methods to enhance cancer diagnosis and treatment. AI, one of the fastest-growing fields, holds transformative potential for integrating and optimizing these diverse diagnostic modalities [189]. AI is increasingly employed to analyze complex multi-omics data, identify patterns in large datasets, and assist in interpreting genetic information, thereby enhancing the accuracy, efficiency, and overall effectiveness of research [190].

AI algorithms, including deep learning, convolutional neural networks, and support vector machines, are being applied to predict tumor markers, assess real-time treatment responses, and identify new opportunities for personalized oncology [189]. These advancements improve the sensitivity and specificity of liquid biopsy methods, particularly through AI-assisted platforms that analyze ctDNA [191] and cfRNA [192]. By automating and accelerating the analysis process, these platforms enable faster and more accurate detection of early-stage cancers. Furthermore, AI models have the potential to combine liquid biopsy data with imaging findings, providing a more comprehensive and precise diagnosis of cancer and its progression [181]. The integration of AI with liquid biopsy could lead to the development of clinically applicable predictive models, enabling more precise, individualized treatment strategies tailored to the unique molecular profile of each patient.

As we continue to advance in this field, it is essential to address ethical considerations surrounding patient data privacy and the potential misinterpretation of genetic information. The development of AI-based diagnostic tools must be accompanied by clear guidelines on data usage and security. Ensuring the accessibility of these technologies in high- and low-resource settings is essential to broaden their impact and address disparities in cancer care [193].

The clinical application of liquid biopsy necessitates large-scale validation before it can be adopted as routine practice. Although studies in other cancers have shown promising results [194,195,196,197], EC presents unique challenges that require dedicated clinical trials. Large-scale validation is crucial for transitioning liquid biopsy into routine clinical use. We advocate for EC-specific trials to confirm the clinical utility of these innovations and establish new standards that improve prognosis and quality of life for EC patients.

## 6. Conclusions

Liquid biopsy is a minimally invasive and effective tool for cancer management, enabling real-time molecular profiling of tumors and capturing their dynamic complexity. Its ability to allow repeat sampling makes it especially valuable for monitoring tumor progression, particularly when traditional biopsies are not feasible. While liquid biopsy has demonstrated clinical utility in other cancers and is already integrated into practice, its application in EC is only now gaining broader recognition.

Despite the promising prospects of liquid biopsy for EC management, several significant limitations currently hinder its clinical implementation. First, the technical challenges include low sensitivity for low-grade tumors and low ctDNA detection rates in early-stage disease [104,116,198] compounded by tumor heterogeneity and the short half-life of ctDNA fragments [45]. Clinical implementation is further impeded by the absence of standardized protocols [199], limited validation, high costs, requirements for specialized training and advanced laboratory infrastructure, and regulatory approval gaps [200]. Disease-specific challenges, including the accessibility of endometrial tumors through traditional biopsy methods, which reduces the immediate need for liquid biopsy, as well as the complexity of multiple molecular subtypes that require comprehensive biomarker panels, lead to higher technical demands and costs [201]. Addressing these challenges through technological advancements and standardization is essential to unlock the full potential of liquid biopsy in clinical practice. Meanwhile, expanding research and conducting large-scale clinical trials are essential to validate its effectiveness and clarify its role in patient care. Taken together, these efforts are essential to unlock the full potential of liquid biopsy, paving the way for more personalized, precise, and effective treatment strategies in EC.

## Figures and Tables

**Table 1:** Application of liquid biopsy in blood samples.

Biomarkers	Detection Method	No. of Participants (EC/Control)	Clinical Significance/Findings/Accuracy	Author and Year	
CTCs					
TOPO48 AAb, Survivin-expressing CCC	ELISA, RT-PCR–ELISA	80/80	The combination of TOPO48 AAb and survivin-expressing CCC improves early diagnosis (93.3% sensitivity) and prognostic stratification (survival outcomes) in early-stage EC. AUC: 0.927 (0.871–0.984) for combined biomarkers; Sensitivity: 74.5% (TOPO48 AAb); specificity: 100% (TOPO48 AAb)	Jiang et al., 2019 [100]	
ANXA2	qPCR and high-throughput screening	57 EC	ANXA2 expression in CTCs predicts EC recurrence and progression. Daunorubicin was identified as inhibiting ANXA2+ tumor cells.	Herrero et al., 2021 [101]	
ER	CellSearch^®^ System	10 Stage I–II EC	CTCs were detected in ovarian vein samples (8/10 patients) during surgery but not in peripheral blood samples. The potential prognostic value for recurrence risk requires validation in a larger cohort.	Francini et al., 2023 [102]	
Pan-CK, GATA3, HER2, HE4, CD13	V-BioChip microfluidic device	8 EC/9 other cancers	EC patients had preoperative expressions of all four markers. CD13 was identified as an alternative prognostic marker for both cervical and CE.	Law et al., 2023 [103]	
cfDNA or ctDNA					
PTEN, KRAS, CTNNB1, PIK3CA	NGS	48 EC	Mutations in plasma were significantly associated with advanced stage, deep myometrial invasion, lymphatic/vascular invasion, and larger tumor size.	Bolivar et al., 2019 [104]	
CfDNA, RTL	qRT-PCR	40/31	cfDNA RTL analysis may be a diagnostic tool for EC detection at an early stage, while its diagnostic performance seems unsatisfactory for cancer progression, staging, and grading. AUC (95% CI): 0.87 (0.79–0.95); sensitivity (95% CI): 80.0% (64.35–90.95%); specificity (95% CI): 80.65% (62.53–92.55%)	Benati et al., 2020 [105]	
Low-molecular-weight cfDNA	Fluorometric quantification	91/22	The concentration of LMW cfDNA was significantly higher in women with uterine cancer and associated with advanced stage, aggressive histology, and worse OS.	Gressel et al., 2020 [106]	
PIK3CA, KRAS	ddPCR	199 EC	ctDNA detection in pre-operative plasma was linked to advanced FIGO stage, aggressive histology, LVSI, and shorter RFS and OS.	Shintani et al., 2020 [107]	
TEPs RNA, ctDNA	RNA-Seq and DNA sequencing	53 EC, 38 benign gynecologic conditions, 204 healthy	ctDNA and TEPs presented the potential for EC diagnosis and tumor histology evaluation preoperatively. TEPs AUC: 97.5% (vs. healthy), 84.1% (vs. benign); ctDNA AUC: 96% (tumor tissue); 69.8% (blood). CtDNA sensitivity: 77.8%; CtDNA specificity: 58%	Łukasiewicz et al., 2021 [14]	
PTEN, TP53, FAT4, ARID1A, ZFHX3, ATM, FBXW7	ddPCR	9 EC	Post-operative ctDN A detection predicted tumor relapse. DFS was shorter for ctDNA-positive cases. AUC: N/A; sensitivity: 100%; specificity: 83.3	Feng et al., 2021 [108]	
Tumor-specific DNA junctions	qPCR	11 EC	Pre-surgical ctDNA was detected in 60% (6/10) and correlated with advanced stage and aggressive disease features. Post-surgical ctDNA detected in 27% (3/11), 2/3 experienced recurrence.	Grassi et al., 2021 [109]	
ZSCAN12, OXT	Methylation-specific ddPCR	Retrospective: 108 tumor tissues; prospective: 33/55	ZSCAN12 and OXT methylation in plasma offered high specificity and sensitivity for EC prediction. AUC: 0.99; sensitivity: 98%; specificity: 97%	Beinse et al., 2022 [110]	
DNMT3A, TET2, and others	NGS	21 EC	A poorer prognosis may be correlated with mutations related to ARCH (DNMT3A and TET2).	Kodada et al., 2023 [111]	
129 genes with molecular barcoding	NGS	44 EC	Presence of ctDNA at baseline or post-surgery was significantly associated with reduced PFS. Correlation with disease stage, progression, and treatment response.	Ashley et al., 2023 [112]	
16 somatic single nucleotide variants (SNVs)	mPCR-NGS	101 Stage I uterine malignancies (88% EC)	Post-surgical ctDNA detection is prognostic of poor RFSin patients with Stage I EC.	Recio et al., 2024 [113]	
TP53, DNMT3A, PIK3CA, PTEN, ERBB2, CTNNB1, PPP2R1A	NGS	61 EC	cfDNA sequencing in advanced EC provided 90% informative results and 87.5% accuracy in molecular subclassification.	Blanc-Durand et al., 2024 [114]	
TP53, PIK3CA, PTEN, ARID1A, KRAS, CCNE1, ERBB2, FBXW7	Hybrid-capture NGS for SNVs, indels, CNVs, fusions, MSI, bTMB	1988 advanced/recurrent EC	TP53 mutations associated with worse OS.	Pina et al., 2024 [115]	
PTEN, PIK3CA, TP53, ARID1A, KRAS, CTNNB1, PIK3R1, FBXW7, PPP2R1A, FGFR2	ddPCR, targeted sequencing, qubit fluorometry	198 EC	High pre-surgery cfDNA and detectable ctDNA correlate with poor DFS and DSS.	Casas-Arozamena et al., 2024 [116]	
TP53, PIK3CA, PTEN, KRAS, CTNNB1, AKT1, BRAF, ERBB2	NGS	24 EC, 17 OC, 2 synchronous endometrial/ovarian carcinomas, 1 endocervical adenocarcinoma	Preoperative ctDNA detection was associated with advanced stage, elevated CA125, and recurrence.	Jamieson et al., 2025 [117]	
cfRNA or ctRNA					
lncRNA DLEU1	RT-qPCR	128/50 endometrial hyperplasia/50 controls	Higher lncRNA DLEU1 levels were associated with advanced clinicopathological features and worse overall and DFS in EC patients. AUC (95% CI): [EC vs. controls: 0.883 (0.826–0.926), EC vs. hyperplasia: 0.766 (0.697–0.826)]; sensitivity: [EC vs. controls: 77.3%, EC vs. hyperplasia: 60.9%]; specificity: [EC vs. controls: 92.0%, EC vs. hyperplasia: 90.0%]	Shan et al., 2020 [118]	
miR-20b-5p, miR-143-3p, miR-195-5p, miR-204-5p, miR-423-3p, miR-484	qRT-PCR	92/102	The 6-miRNA signature demonstrated very consistent diagnostic performance in three datasets across cohorts. AUC: [training: 0.748, testing: 0.833, external validation: 0.967]; sensitivity: [training: 78.4%, testing: 77.1%, external validation: 83.3%]; specificity: [training: 63.0%, testing: 66.7%, external validation: 100%]	Fan et al., 2021 [119]	
miR-204-5p	RT-qPCR	52/60	Metastasis of lymph nodes was associated with downregulation of serum miR-204-5p. AUC (95% CI): 0.923 (0.847–1.000); sensitivity: 87.2%; specificity: 80%	Wu et al., 2022 [120]	
miRNA133a-2, miRNA-21, miRNA-205	qRT-PCR	36/15	These miRNAs could serve as potential prognostic biomarkers for endometrial carcinoma.	Salim et al., 2022 [121]	
miR-16, miR-99b, miR-20a, miR-145, miR-143, miR-125a	qRT-PCR	10/10	miR-16, miR-99b, miR-125a, and miR-145 could serve as diagnostic indicators for endometrioid EC. AUC: 0.957 (miR-145); sensitivity: 90% (miR-145); specificity: 100% (miR-145)	Kumari et al., 2023 [122]	
miR-155-5p, miR-200b-3p, miR-589-5p, and others	Small RNA sequencing	316/316	These RNAs hold potential as early biomarkers for EC, which could facilitate timely interventions. Relationships between EC and miRNAs were modified by body mass index, physical activity, and smoking status.	Rostami et al., 2024 [123]	
EVs					
LGALS3BP	TMT labelling, ELISA	87 EC/12 AEH/42 controls	Plasma exosomal LGALS3BP levels correlated with EC progression and poor prognosis. AUC (95% CI): 0.7406 (0.6506–0.8305)	Song et al., 2020 [124]	
miR-15a-5p, miR-106b-5p, miR-107	ddPCR	115/87	Exosomal miR-15a-5p was highly predictive of the aggressiveness and p53 mutation status of EC tumors and markedly elevated in early-stage EC. AUC: 0.813 (miR-15a-5p); 0.899 miR-15a-5p combined serum tumor markers (CEA and CA125)	Zhou et al., 2021 [125]	
APOA1, HBB, CA1, HBD, LPA, SAA4, PF4V1, APOE	LFQ-MS	36/36	Eight significantly upregulated proteins were identified in serum exosomes, indicating potential as early-stage EC biomarkers. AUC (95% CI): 0.98 (0.95–1) (Stage 1 EC); sensitivity: 100% (Stage 1 EC); specificity: 86.11% (Stage 1 EC)	Sommella et al., 2022 [126]	
Proteomics					
CFB, TF, CAT, PSMB6, B2M, PCDH18	HPLC-MS/MS	112/112	Six proteins could distinguish EC cases from the control group, with the strongest performance ≤ 2 years pre-diagnosis. AUC (95% CI): 0.72–0.88; sensitivity: 45.2% (cutoff: 0.5); specificity: 96.4% (cutoff: 0.5)	Tarney CM et al., 2019 [127]	
CLU, SERPINC1, ITIH4, C1RL, APOC3, DSG1	2D-DIGE, WB, LC-MS/MS	15/15	The study identified 16 proteins with diagnostic potential for EC. Validation showed upregulation of CLU, ITIH4, SERPINC1, and C1RL in EC serum and exosomes. AUC: 0.9289; sensitivity: 100%; specificity: 86.67%	Ura et al., 2021 [128]	
Gal-1, Gal-9, MMP7, FASLG, COL9A1	Proximity extension assay (PEA)	44/44	Combined proteins from the Immuno-oncology panel and the Target 96 Oncology III panel showed differential expression in early-stage Type I EC with high diagnostic accuracy AUC (95% CI): 0.969 (0.939–0.999); sensitivity: 97.67%; specificity: 83.72%	Ura et al., 2022 [129]	
Suprabasin (SBSN) (isoforms 1 & 2)	2D-DIGE and MS, validated by WB	Proteomic: 10/10, validation: 30/30 (serum), 30/30 (tissue)	In serum or tissue, SBSN, particularly isoform 2, may be a novel biomarker for EC. AUC: [isoform 2 (serum): 0.75, (tissue): 0.79]	Celsi et al., 2022 [130]	
FABP-1, α-2 macroglobulin, ZAG, Ero1-α, haptoglobin, and others	2D-DIGE, MALDI-TOF-MS	8 diabetic EC/8 non-diabetic EC	Downregulation of FABP-1 and haptoglobin, and upregulation of ERO1-α, α-2-macroglobulin, and ZAG in EC with diabetes indicated severe disease and poor prognosis.	Mujammami et al., 2024 [131]	
Metabolomics					
183 metabolites	LC-MS	40 EC	Metabolite patterns were associated with survival. Methionine sulfoxide elevation was linked to poor prognosis. AUC: [Model 3: 0.965 (0.913–1)]	Strand et al., 2019 [132]	
268 serum metabolites	GC-MS	Training: 120 (50/70), validation: 1430	The EC screening of postmenopausal women using an ensemble EML algorithm achieved an accuracy rate of > 99%. Sensitivity: 100%; specificity: 99.86%	Troisi et al., 2020 [133]	
17-OHP, 11-DOC, A4, E1, E2	LC-MS/MS	100 EC	Low levels of 17-OHP, 11-DOC, and A4 were associated with aggressive EC phenotypes and poor disease-specific survival.	Forsse et al., 2020 [134]	
Ceramides, acylcarnitines, 1-methyladenosine	HPLC-TQ/MS	15/21	The combined panel was identified as superior to individual biomarkers for early disease detection. AUC (95% CI): 0.925 (0.905–0.945); sensitivity: 94%; specificity: 75%	Kozar et al., 2021 [135]	
Phospholipids, sphingolipids	MS	67/69	Lipid metabolites were effectively discriminated EC in women with BMI ≥ 30 kg/m^2^. AUC: 0.95	Njoku et al., 2021 [136]	
Amino acids, sphingolipids, carnitine	LC-MS/MS	853/853	Identified metabolites were associated with EC risk.	Dossus et al., 2021 [137]	
Pregnenolone, progesterone, 17-hydroxypregnenolone, and others	LC-MS/MS	EC: 65/345; OC: 67/413	17-Hydroxypregnenolone was inversely associated with EC risk and positively associated with ovarian cancer risk.	Trabert et al., 2021 [138]	
6-keto-PGF1α, PA (37:4), LysoPC (20:1), PS (36:0)	UPLC-Q-TOF/MS	326/225	Specific biomarkers for endometrial polyps were identified to distinguish them from EC or hyperplasia. AUC: [EP vs. EC: 0.915; EP vs. EH: 1.000]; sensitivity: [EP vs. EC: 100%; EP vs. EH: 100%]; specificity: [EP vs. EC: 72.41%; EP vs. EH: 100%]	Yan et al., 2022 [139]	
Leptin, IL-8, sTie-2, follistatin, neuropilin-1, G-CSF	Luminex xMAP™ Multiplexing Technology	91/111	Leptin was significantly higher in EC patients, especially in Type 1 EC. IL-8 levels were elevated in Type 2 EC and poorly differentiated G3 tumors and those with vascular invasion. AUC: [training: 0.94; testing: 0.81]	Roškar et al., 2022 [140]	
117 metabolites	LC-MS/MS, FIA-MS/MS	1706 EC	An inverse association between EC risk and a glycine/serine metabolite cluster was found.	Breeur et al., 2022 [141]	
Ursodeoxycholic acid, PC (O-14:0_20:4), Cer (d18:1/18:0)	UHPLC-MS/MS	Discovery: 18/20, validation: 20 EC/20 atypical endometrial hyperplasia	Lipid biomarkers differentiated early-stage EC from healthy controls and AEH patients. AUC: [discovery: 0.903; validation: 0.928]; sensitivity: [discovery: 83.3%; validation: 85%]; specificity: [discovery: 85%; validation: 85%]	Cheng et al., 2023 [142]	
11-oxygenated androgens (11KAST, 11OHAST, etc.)	LC-MS/MS	272 EC	Higher preoperative free 11KAST and postoperative 11OHAST levels were associated with increased risk of recurrence and poor DFS.	Dahmani et al., 2023 [143]	
LysoPC, TGs, amino acids	UHPLC-MS/MS	142/154	Histidine and tryptophan levels decreased with disease progression and recurrence risk. AUC: [top 5 metabolites: 0.997 (0.986–1)]	Hishinuma et al., 2023 [144]	
338 metabolites	LC-HRMS	20 EC, 20 hyperplasia, 19 controls	Plasma metabolic signatures distinguished EC and hyperplasia from healthy controls. AUC: 0.821 [15 metabolic variations]	Benabdelkamel et al., 2024 [145]	
Multi-omics					
Metabolites and lncRNAs	LC-MS/MS, LncRNA sequencing	Endometrial dysplasia: 4, Stage I EC: 4, Stage III EC: 4, controls: 4	Metabolites and lncRNAs correlated with EC progression. AUC: 2,3-pyridinedicarboxylic acid: 0.69, hematommic acid, ethyl ester: 0.69, maltitol: 0.69, 13 (S)-HODE: 0.88, D-mannitol: 0.69	Hao et al., 2023 [146]	
Various metabolites and proteins	GWAS and Mendelian randomization	121,885 participants (12,906 EC)	Key metabolites and proteins influenced EC subtypes.	Shen et al., 2024 [147]	
CTCs, lncRNAs, and DNA methylation markers	Microfluidic CTC isolation, RT-qPCR, MSP/qMSP	71/14	Combined biomarkers improved diagnostic accuracy for EC compared to individual biomarkers alone. AUC (95% CI): 0.94 (0.89–0.98); sensitivity (95% CI): 89% (82–94%); specificity (95% CI): 92% (85–96%)	Ding et al., 2024 [148]	

**Table 2:** Application of liquid biopsy in urine samples.

Category of Liquid Biopsy	Biomarkers	Detection Method	No. of Participants (EC/Control)	Clinical Significance/Findings/Accuracy	Author and Year
Proteomics	CDH1, VTN, HSPG2	Nano HPLC-ESI-MS/MS	5/7	Downregulation of key proteins suggested potential urinary biomarkers for early detection of EC.	Kacírová et al., 2019 [152]
miRNA	miR-3973; -4426; -5089-5p and -6841	RT-qPCR	10/30	These biomarkers served as promising candidates for urine-based liquid biopsies in detecting EC.	Ritter et al., 2020 [153]
cfDNA	47-gene panel (POLE, TP53)	NGS	19/20	Evaluating urine for somatic mutations offered a non-invasive, accurate approach for detecting EC and molecular classification. AUC: 0.99; sensitivity (95% CI): 100.0% (82.4–100.0%); specificity (95% CI): 95.0% (75.1–99.9%)	Costas et al., 2023 [154]
Proteomics	SPRR1B, CRNN, CALML3, TXN, FABP5, C1RL, MMP9, ECM1, S100A7, CFI	SWATH-MS with ML	50/54	EC patients discriminated from symptomatic controls suggested its potential as a non-invasive diagnostic tool. AUC (95% CI): 0.92 (0.86–0.97); sensitivity: 83.7%; specificity: 83.9%	Njoku et al., 2023 [155]
Metabolomics	Baicalin, 5beta-1,3,7 (11)-eudesmatrien-8-one, indolylacryloylglycine, edulitine, physapubenolide	UPLC-MS	42 EC (22 PT/20 CR)	The predictive biomarkers presented great potential diagnostic value in fertility-sparing treatments for EC patients. AUC: [training: 0.982, validation: 0.851]; sensitivity: [training: 97.5%, validation: 86.4%]; specificity: [training: 96.7%, validation: 90.0%]	Chen et al., 2023 [156]
Metabolomics	ADP-mannose, docosatrienoic acid, hippuric acid	UPLC-MS	146/59	Combined urine–serum metabolomics effectively distinguished EC from controls, high-risk from low-risk EC, and Type I vs. II EC. AUC: [training: 0.953; validation: 0.972]; sensitivity: [training: 0.857; validation: 0.846]; specificity: [training: 0.876; validation: 0.974]	Chen et al., 2024 [157]
Metabolomics and transcriptomics	10 metabolites (histamine, 1-methylhistamine, methylimidazole acetaldehyde, etc.) and 3 hub genes (RRM2, TYMS, TK1)	LC-MS	110/110	The combination of these biomarkers demonstrated enhanced diagnostic accuracy compared to individual markers. AUC: combined: 0.90; sensitivity: combined: >0.85; specificity: combined: >0.85	Fu et al., 2024 [158]

**Table 3:** Application of liquid biopsy in other samples.

Category of Liquid Biopsy	Biomarkers	Detection Method	No. of Participants (EC/Control)	Clinical Significance/Findings/Accuracy	Author and Year
Uterine lavage fluid/uterine aspirates					
cfDNA, CTCs	PTEN, PIK3CA, TP53, CTNNB1, KRAS, etc.	NGS, ddPCR, CellSearch system	60 EC	Genetic alterations were detected in 93% of EC through UAs. ctDNA was associated with high-risk tumors and disease progression.	Casas-Arozamena et al., 2020 [159]
cfDNA	BAT26, BAT25, NR24, NR21, Mono27	ddPCR	90 EC	A high concordance (96.67%) between MSI determinations in cfDNA and the standard of care was confirmed.	Casas-Arozamena et al., 2023 [160]
cfRNA	miR-146a-5p, miR-183-5p, miR-429	Real-time PCR	42/40	miR-146a-5p, miR-183-5p, and miR-429 were significantly upregulated in EC. AUC: miR-183-5p: 0.675, miR-429: 0.709, miR-146a-5p: 0.685	Yang et al., 2023 [161]
Cervicovaginal fluid/cervicovaginal lavage					
Metabonomics	Phosphocholine, malate, asparagine	NMR spectroscopy	21/33	Metabolomic biomarkers in CVF for non-invasive detection of EC were identified and validated using ML algorithms. AUC: [training: 0.88–0.92; test: 0.75–0.80]; sensitivity (95% CI): forests: 0.75 (0.19–0.99); specificity (95% CI): forests: 0.80 (0.28–1.00)	Cheng et al., 2019 [162]
Cytology	Malignant endometrial cells	Cytological analysis	103/113	Vaginal cytology demonstrated higher sensitivity (90.2%) compared to urine cytology (72.0%) but lower specificity. Sensitivity: [vaginal: 90.2%, urine: 72.0%, combined: 91.7%]; specificity: [vaginal: 88.7%, urine: 94.9%, combined: 88.8%]	O’Flynn et al., 2021 [163]
Proteomics	72 proteins (TIM-3, VEGF, TGF-α, IL-10, CA19–9, CA125, etc.)	Multiplex immunoassays	66/126	Identified lavage proteins could discriminate EC from benign conditions. AUC (95% CI): combined: 0.91 (0.78–0.97) Sensitivity: 86.1% (combined); specificity: 87.9% (combined)	Łaniewski et al., 2022 [164]
Metabolomics and proteomics	Amino acid and nucleotide metabolism biomarkers	LC-MS/MS	44/43	Urine/intrauterine brushing metabolites correlate with tissue pathways (amino acid/nucleotide metabolism). AUC: 0.808 (urine) 0.847 (intrauterine brushing); Sensitivity: urine: 74.7% (top 5 metabolites)	Yi et al., 2022 [165]
Somatic mutations	47 genes panel (POLE, TP53, PTEN, etc.)	NGS	139/107	POLE mutations indicated excellent prognosis; TP53 mutations were associated with significant DFS differences among molecular subtypes. AUC: 0.83 (self-collected); sensitivity: 73% (clinician and self-collected); specificity: [80% (clinician-collected), 90% (self-collected)]	Pelegrina et al., 2023 [166]
DNA methylation	ZSCAN12, GYPC	WID-qEC	12/375	WID-qEC test demonstrated superior diagnostic accuracy compared to transvaginal ultrasound in detecting uterine cancers. AUC (95% CI): 0.943 (0.847–1.000); sensitivity (95% CI):90.9% (62.3–98.4); specificity (95% CI): 92.1% (88.9–94.4)	Evans et al., 2023 [167]
Proteomics	SERPINH1, VIM, TAGLN, PPIA, CSE1L, CTNNB1	MS	22/19	Six protein biomarkers in cervical fluids were identified to distinguish women with abnormal uterine bleeding who are EC and those who are non-EC. AUC: [UF: > 0.71, LDHA, ENO1, PKM: > 0.9; M1: up to 0.83 (SERPINH1); M3: up to 0.84 (TAGLN)]; sensitivity: [M1: up to 83%; M3: up to 89%]; specificity: [M1: up to 81%; M3: up to 78%]	Martinez-Garcia et al., 2023 [168]
DNA methylation	ZSCAN12, GYPC	WID-qEC	28/74	The WID-qEC test reliably detected uterine cancers (endometrial and cervical) across sampling devices and collection methods (gyn. vs. patient self-sampling). AUC (95% CI): 0.96 (0.91–1.00); sensitivity: 92.9% (gyn), 75.0% (self); specificity: 98.6% (gyn), 100.0% (self)	Illah et al., 2024 [169]
DNA methylation	CDO1m, CELF4m	qMSP	21/275	Dual-gene methylation showed high sensitivity (85.7%) and specificity (87.6%) for EC screening. AUC (95% CI): 0.867 (0.788–0.946) for dual methylation; sensitivity (95% CI): 85.7% (0.707–1.000); specificity: 87.6% (0.837–0.915)	Zhao et al., 2024 [170]
DNA methylation	CDO1, CELF4	qPCR	40/98	Combined test specificity (95.9%) outperformed transvaginal ultrasound (ET) and CA125 and detected all Type II EC cases. AUC (95% CI): 0.917 (0.853–0.91) for combined test; sensitivity (95% CI): 87.5% (73.2–95.8); specificity: 95.9% (89.9–98.9)	Cai et al., 2024 [171]
Proteomics	HPT, LG3BP, FGA, LY6D, IGHM	SWATH-MS	53/65	Cervico-vaginal fluid protein signatures showed superior accuracy over plasma in detecting Stage I EC and advanced tumors effectively. AUC (95% CI): [cervico-vaginal: 0.95 (0.91–0.98), plasma: 0.87 (0.81–0.93)]; sensitivity: [cervico-vaginal: 91% (83–98%), plasma: 75% (64–86%)]; specificity: [cervico-vaginal: 86% (78–95%), plasma: 84% (75–93%)]	Njoku et al., 2024 [172]
Proteomics	Angiopoietin-2, endoglin, FAP, MIA, VEGF-A	Multiplex immunoassays	66 EC/108 benign	Five key biomarkers were significantly elevated in EC. The multivariate model showed prognostic value for tumor grade, size, invasion, and MMR status. AUC: 0.918; sensitivity: 87.8%; specificity: 90.7%	Harris et al., 2024 [173]
Metabolomics	Lipids, amino acids, and other metabolites	UPLC-MS	66/108	Metabolic dysregulation was linked to tumor characteristics (size, myometrial invasion); noninvasive detection and risk stratification improved; multivariate models achieved high diagnostic accuracy. AUC: 0.800–0.951 (25-feature model); sensitivity: 78.6% (for EC); specificity: 83.3% for EC, 79.6% for benign	Lorentzen et al., 2024 [174]
Tampons					
DNA methylation	28 Methylated DNA markers	qMSP	100/92	The sensitivity to detecting EC was high even when vaginal fluid samples were collected before endometrial sampling. AUC (95% CI): 0.91 (0.85–0.97); sensitivity (95% CI):82% (70–91%); specificity (95% CI): 96% (87–99%)	Bakkum-Gamez et al., 2023 [175]
Cervical scrapings and vaginal swabs					
Genomic DNA	100 EC-related genes	NGS	39/11	Cervical swab-based gDNA genomic data demonstrated enhanced detection ability and enabled patient classification. Sensitivity: 67%; specificity: 100%	Kim et al., 2022 [176]
DNA methylation	BHLHE22, CDO1	MPap	494 EC	MPap test showed high sensitivity and specificity for EC detection. AUC (95% CI): [Stage 1: 0.91 (0.87–0.94), Stage 2: 0.90 (0.84–0.95)]; sensitivity (95% CI): [Stage 1: 92.9% (80.5–98.5%), Stage 2: 92.5% (82.9–100.0%)]; specificity (95% CI): [Stage 1: 71.5% (64.8–77.5%), Stage 2: 73.8% (67.6–79.4%)]	Wen et al., 2022 [177]
DNA methylation	GYPC, ZSCAN12	qPCR	562 (various groups)	The WID-qEC test offered a non-invasive EC screening and triage with high sensitivity and specificity. AUC: 0.94 (Barcelona); sensitivity: [97.2% (FORECEE), 90.1% (Barcelona), 100% (PMB cohort)]; specificity: [75.8% (FORECEE), 86.7% (Barcelona), 89.1% (PMB cohort)]	Herzog et al., 2022 [178]
DNA methylation	ADCYAP1, BHLHE22, CDH13, CDO1, GALR1, GHSR, HAND2, SST, ZIC1	qMSP	103/317	DNA methylation marker analysis in urine, cervicovaginal self-samples, and clinician-taken cervical scrapes achieved high diagnostic accuracy for EC detection. AUC: [urine: 0.95, self-samples: 0.94, scrapes: 0.97]; sensitivity: [urine: 90%, self-samples: 89%, scrapes: 93%]; specificity: [urine: 90%, self-samples: 92%, scrapes: 90%]	Wever et al., 2023 [179]
DNA methylation	RASSF1A, HIST1H4F	qPCR	19/75	Methylation levels of RASSF1A/HIST1H4F increased with endometrial lesion severity. AUC: RASSF1A: 0.938; HIST1H4F: 0.951	Wang et al., 2024 [180]
Other samples					
cfDNA	KRAS, PIK3CA	NGS, qPCR	50/7	KRAS/PIK3CA mutations were detected in 47.4% of peritoneal lavages and correlated with tumor tissue.	Mayo-de-las-Casas et al., 2020 [181]
Metabolomics and proteomics	SOAT1, CE	ELISA, colorimetric assay, RT-qPCR, IHC	32/16	SOAT1 and CE may be associated with malignancy, aggressiveness, and poor prognosis. AUC: peritoneal fluid SOAT1: 0.767; sensitivity: 80%; specificity: 67%	Ayyagari et al., 2023 [182]

## Data Availability

No new data were created or analyzed in this study.
